# Evaluation of the Recovery Engagement and Coordination for Health–Veterans Enhanced Treatment Suicide Risk Modeling Clinical Program in the Veterans Health Administration

**DOI:** 10.1001/jamanetworkopen.2021.29900

**Published:** 2021-10-18

**Authors:** John F. McCarthy, Samantha A. Cooper, Kallisse R. Dent, Aaron E. Eagan, Bridget B. Matarazzo, Claire M. Hannemann, Mark A. Reger, Sara J. Landes, Jodie A. Trafton, Michael Schoenbaum, Ira R. Katz

**Affiliations:** 1 Office of Mental Health and Suicide Prevention, Department of Veterans Affairs, Washington, District of Columbia; 2 Rocky Mountain Mental Illness Research, Education and Clinical Center, Department of Veterans Affairs, Aurora, Colorado; 3 VA Puget Sound Healthcare System, Seattle, Washington; 4 South Central Mental Illness Research Education Clinical Center, Department of Veterans Affairs, Little Rock, Arkansas; 5 National Institute of Mental Health, Bethesda, Maryland

## Abstract

**Importance:**

The Veterans Health Administration (VHA) implemented a national clinical program using a suicide risk prediction algorithm, Recovery Engagement and Coordination for Health–Veterans Enhanced Treatment (REACH VET), in which clinicians facilitate care enhancements for individuals identified in local top 0.1% suicide risk tiers. Evaluation studies are needed.

**Objective:**

To determine associations with treatment engagement, health care utilization, suicide attempts, safety plan documentation, and 6-month mortality.

**Design, Setting, and Participants:**

This cohort study used triple differences analyses comparing 6-month changes in outcomes after vs before program entry for individuals entering the REACH VET program (March 2017-December 2018) vs a similarly identified top 0.1% suicide risk tier cohort from prior to program initiation (March 2014-December 2015), adjusting for trends across subthreshold cohorts. Subcohort analyses (including individuals from March 2017-June 2018) evaluated difference-in-differences for cause-specific mortality using death certificate data. The subthreshold cohorts included individuals in the top 0.3% to 0.1% suicide risk tier, below the threshold for REACH VET eligibility, from the concurrent REACH VET period and from the pre–REACH VET period. Data were analyzed from December 2019 through September 2021.

**Exposures:**

REACH VET–designated clinicians treatment reevaluation and outreach for care enhancements, including safety planning, increased monitoring, and interventions to enhance coping.

**Main Outcomes and Measures:**

Process outcomes included VHA scheduled, completed, and missed appointments; mental health visits; and safety plan documentation and documentation within 6 months for individuals without plans within the prior 2 years. Clinical outcomes included mental health admissions, emergency department visits, nonfatal suicide attempts, and all-cause, suicide, and nonsuicide external-cause mortality.

**Results:**

A total of 173 313 individuals (mean [SD] age, 51.0 [14.7] years; 161 264 [93.1%] men and 12 049 [7.0%] women) were included in analyses, including 40 816 individuals eligible for REACH VET care and 36 604 individuals from the pre–REACH VET period in the top 0.1% of suicide risk. The REACH VET intervention was associated with significant increases in completed outpatient appointments (adjusted triple difference [ATD], 0.31; 95% CI, 0.06 to 0.55) and proportion of individuals with new safety plans (ATD, 0.08; 95% CI, 0.06 to 0.10) and reductions in mental health admissions (ATD, −0.08; 95% CI, −0.10 to −0.05), emergency department visits (ADT, −0.03; 95% CI, −0.06 to −0.01), and suicide attempts (ADT, −0.05; 95% CI, −0.06 to −0.03). Subcohort analyses did not identify differences in suicide or all-cause mortality (eg, age-and-sex-adjusted difference-in-difference for suicide mortality, 0.0007; 95% CI, −0.0006 to 0.0019).

**Conclusions and Relevance:**

These findings suggest that REACH VET implementation was associated with greater treatment engagement and new safety plan documentation and fewer mental health admissions, emergency department visits, and suicide attempts. Clinical programs using risk modeling may be effective tools to support care enhancements and risk reduction.

## Key Points

**Question:**

Is the Veterans Health Administration Recovery Engagement and Coordination for Health–Veterans Enhanced Treatment (REACH VET) program, which facilitates care enhancements for individuals in the top 0.1% suicide risk tier using a validated algorithm, associated with health care utilization, treatment engagement, suicide attempts, suicide safety plan documentation, and suicide mortality?

**Findings:**

In this cohort study including 173 313 individuals before and after implementation of the REACH VET program using triple differences, inclusion in the REACH VET program was associated with having more outpatient encounters, increased documentation of new suicide prevention safety plans, and fewer inpatient mental health admissions, emergency department visits, and documented suicide attempts.

**Meaning:**

These findings suggest that clinical programs using predictive modeling can support care enhancements and risk reduction.

## Teaser

This cohort study examines treatment engagement, health care utilization, and suicide-related outcomes associated with the Veterans Health Administration's Recovery Engagement and Coordination for Health–Veterans Enhanced Treatment (REACH VET) suicide risk modeling clinical program.

## Introduction

Veteran suicide rates exceed those of other US adults.^1,2^ Suicide prevention is the top clinical priority of the Department of Veterans Affairs (VA).^3,4,5^ Informed by prior work,^6,7^ VA and National Institute of Mental Health scientists developed an expansive suicide mortality risk prediction algorithm using Veterans Health Administration (VHA) electronic health records.^8^ They identified individuals at risk rather than individual risk factors. Patients in the top 0.1% tier of risk died from suicide at a rate 30-fold higher than the overall VHA patient population.^8^ Machine learning analyses^9^ generated prediction similar to that of the proof-of-concept model^8^ using fewer variables, facilitating further targeted efforts^10^ to complement clinical assessments.

In 2017, the VHA began national implementation of the Recovery Engagement and Coordination for Health–Veterans Enhanced Treatment (REACH VET) program, applying the algorithm^9^ to identify individuals in facilities’ top 0.1% suicide risk tiers.^11^ Using an interactive dashboard, program coordinators receive information and communicate with clinicians who re-evaluate treatment strategies and conduct outreach to collaboratively initiate care enhancements. Reevaluation includes assessment of service needs, and clinicians ensure that patients can access services. Treatment enhancements include suicide prevention safety planning, enhanced communication, increased monitoring of stressful events, and interventions to support coping. To facilitate fidelity, actions are documented using templated notes.

Focused outreach and engagement through REACH VET may affect health care processes,^12^ access,^13^ quality, health behavior,^14^ and suicide-related behavior. The program may enhance engagement in evidence-based treatment and treatment adherence; affect health behaviors by increasing the perceived benefits of treatment seeking and adherence, trouble-shooting barriers, and providing cues to action; and address access barriers and enhance quality via improved resources and processes (eg, treatment review, follow-up). These may result in better outcomes, including reduced suicide attempts and deaths.

Little is known regarding program effectiveness. It is important to evaluate outcomes associated with the REACH VET program.^15^ We assess associations with treatment engagement, care processes, health care utilization, and mortality outcomes. We hypothesized that inclusion in REACH VET is associated with enhanced engagement (ie, more scheduled and completed appointments, fewer missed appointments, and more outpatient mental health encounters) and quality of care (ie, suicide safety plans), reduced acute care needs (ie, fewer inpatient mental health admissions and emergency department [ED] visits), and reduced suicide-related behavior (ie, suicide attempts and suicide deaths) and nonsuicide external cause and all-cause mortality.

## Methods

This study was conducted as part of ongoing VHA operations and program evaluation and approved by the Office of Mental Health and Suicide Prevention; it was not classified as research and was exempt from VHA institutional review board review and informed consent. This study is reported following the Strengthening the Reporting of Observational Studies in Epidemiology (STROBE) reporting guideline.

### Data Sources and Measures

The predictive model includes sociodemographic, diagnosis, health care utilization, medication, and prior suicide attempt indicators. Coded into VHA’s Business Intelligence platform, the risk prediction algorithm draws from the VHA Corporate Data Warehouse and is processed monthly for all individuals with VHA inpatient or outpatient care in the prior 2 years, identifying those entering top 0.1% suicide risk tiers at each of 141 VHA administrative parent facilities (service points with shared administrative responsibility).

Measures were assessed from Corporate Data Warehouse except where specified. These examined treatment engagement (ie, non–same-day outpatient appointments, non–same-day completed outpatient appointments, and percentage of scheduled appointments resulting in missed encounters); processes (ie, documentation of suicide prevention safety plan, documentation within 6 months among individuals without plans in the prior 2 years); health care utilization (ie, outpatient mental health visits, inpatient mental health admissions, and ED visit days); documentation of nonfatal suicide attempts (per site reports in the Suicide Prevention Applications Network and the Suicide Behavior and Overdose Report^16^); 6-month mortality per the Vital Status File and all-cause, suicide, and nonsuicide external cause mortality per the VA/Department of Defense Mortality Data Repository of National Death Index search results through 2018; and implementation indicators from operations systems. To enhance sensitivity, health care utilization measures assessed counts rather than some vs none.

### Parallel Trend Assumption: Diagnostics

Preliminary to statistical analyses, for outcomes measured in the prior and subsequent 6 months, we tested the parallel trend assumption for difference-in-differences analyses (eAppendix in the Supplement). Outcomes were collected across four 6-month periods prior to cohort entry. Parallel trends between the REACH VET era’s top 0.1% risk tier and subthreshold cohorts were assessed using an interaction between time period and group. For top 0.1% risk tier cohorts, parallel trends were assessed using interaction between time period and era. The parallel trend assumption failed for outcomes other than completed appointments, scheduled appointments, and proportion of appointments missed, validating use of the triple differences (TD) design. We similarly evaluated residual trends after adoption of the TD approach. Significant preidentification trends were observed for all except for appointment-related outcomes. Trends were close to zero and must be interpreted in the context of main analysis results (eAppendix in the Supplement).

### Design

With an intention-to-treat framework, we examined correlates of REACH VET entry. Pre-post comparisons were necessary because health care utilization measures were elements of the algorithm, and individuals entering the top risk tier could return to lower-risk states as regression to the mean. We evaluated differential change, comparing differences in changes for the REACH VET cohort vs a comparable cohort identified prior to program initiation. We used a TD design to isolate potential program influences from period and cohort trends (Figure).^17^ We compared changes 6 months after vs 6 months before cohort entry for REACH VET and pre–REACH VET top 0.1% suicide risk cohorts, adjusting for differences in pre-post differences for subthreshold risk cohorts (top 0.3%-0.1% tiers) in the REACH VET and pre–REACH VET eras. Inclusion of subthreshold cohorts enabled adjustment for period differences. Tiers were evaluated for patients’ local VHA facilities.

**Figure.**
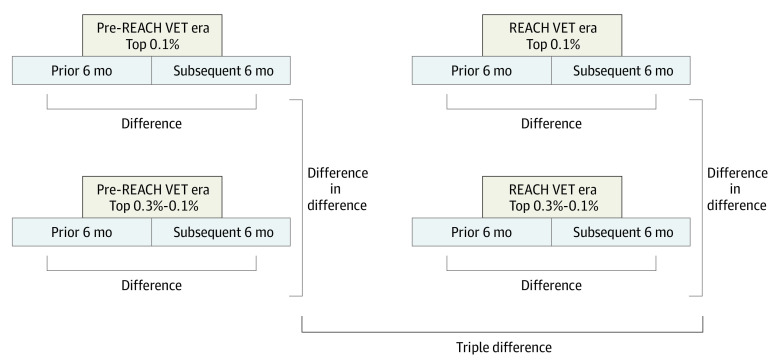
Triple Differences Analysis Design The pre–Recovery Engagement and Coordination for Health–Veterans Enhanced Treatment (REACH VET) era cohorts included individuals identified in the specified risk tier between March 2014 and December 2015; the REACH VET–era cohorts included individuals identified in the specified suicide risk tier between March 2017 and December 2018. Top 0.1% risk tier cohorts included all individuals identified in the top 0.1% tier of suicide risk scores at their facilities. Top 0.3% to 0.1% risk tier cohorts included all individuals identified in the top 0.3% to 0.1% suicide risk tier at their facility and not identified in the top 0.1% risk tier in the 6 months prior to or following identification. Triple differences tests were used for measures assessed in both the 6 months prior to and the 6 months following the individual’s index date.

The TD design examined health care utilization, engagement, suicide safety plan documentation, and nonfatal suicide attempts measures assessed in the prior and subsequent 6 months. For outcomes assessed only after cohort entry (ie, new safety plan documentation, mortality), difference-in-difference analyses compared 6-month outcomes for the top 0.1% risk tier and subthreshold cohorts across the REACH VET implementation and preimplementation periods.

### Cohorts

Program implementation involved training and development. After several months of piloting, in February 2017, the VHA began monthly dissemination of information regarding individuals in top 0.1% risk tiers, with enhanced program dashboards.^18^ We assessed measures for individuals who entered a REACH VET top 0.1% risk tier cohort in March 2017 to December 2018, excluding those identified during initial development (November 2016-February 2017).

Pre–REACH VET comparison cohorts were generated by applying the algorithm monthly for March 2014 to December 2015. Paralleling the implementation period, these cohorts excluded individuals in a top 0.1% risk tier within 4 months prior to March 2014.

For both periods, inclusion required VHA encounters in the prior 2 years. Six-month postidentification periods (180 days) began when individuals were identified as being in a top 0.1% risk tier. Subthreshold cohorts were generated for top 0.3% to 0.1% risk tiers, excluding individuals who were identified in a top 0.1% risk tier prior to or within 6 months after their subthreshold cohort identification date. Cohorts excluded 43 records owing to missing age or sex data and 456 records owing to death prior to their cohort entry date.

### Outcomes by Program Exposure

For measures assessed in both the previous and subsequent 6 months, TDs were calculated using generalized estimating equations at the individual level, with repeated measures and a robust sandwich estimator covariance structure. The TD approach was needed to account for period and cohort trends that violated the difference-in-differences parallel trend assumption. The key variable was a 3-way interaction among subsequent 6-month period (vs prior 6 months), REACH VET era cohort (vs pre–REACH VET cohort), and top 0.1% risk tier (vs top 0.3%-0.1% risk tier). The 3-way interaction was calculated as: *Outcome* = (β_0_ + β_1_ × *Post6Months* + β_2_ × *REACH_VET_Era* + β_3_ × *Top0.1%* + β_4_ × *Post6Months* × *REACH_VET_Era* + β_5_ × *Post6Months* × *Top0.1%* + β_6_ × *REACH_VET_Era* × *Top0.1%* + β_7_ × *Post6Months* × *REACH_VET_Era* × *Top0.1%* + ε_i_). This interaction measures differential changes in the REACH VET era between individuals identified for the program (top 0.1% risk tier) vs those who were not, isolating the program’s associations while controlling for period and cohort confounders. Models included interactions between the grouping variables (ie, prior vs subsequent 6 months, REACH VET vs pre–REACH VET periods, and top 0.1% vs top 0.3%-0.1% risk tiers). Adjusted models controlled for age and sex on the individual’s cohort entry date.

Count outcome models used the Poisson distribution; binary outcome models used the binomial distribution. When binomial models failed to converge, a modified Poisson was used.^19^ For percentage of missed appointments, cohorts were specific to individuals with appointments in both prior and subsequent periods, and models used the normal distribution and identity-link function. In difference-in-differences models, the key variable was the interaction between period and risk tier.

When overdispersion or zero inflation was suspected for count outcomes, negative binomial and zero inflation models were calculated to control for these issues, respectively; these did not change conclusions. We calculated absolute differences using the SAS NLEstimate macro (SAS Institute). Significance was assessed with 2-tailed χ^2^ tests, with α = .05. Analyses used SAS Enterprise Guide, versions 7.1 and 8.2.

For exploratory analyses using cause of death data through 2018, we identified a REACH VET subcohort who qualified between March 2017 to June 2018 and a concurrent subthreshold subcohort. We assessed 6-month all-cause, suicide, and nonsuicide external cause mortality. Pre–REACH VET top risk tier and subthreshold cohorts were included in these analyses. Data were analyzed from December 2019 through September 2021.

## Results

A total of 173 313 individuals (mean [SD] age, 51.0 [14.7] years; 161 264 [93.1%] men and 12 049 [7.0%] women) were included in analyses, including 40 816 individuals in the top 0.1% risk tier from the REACH VET period, and 36 604 individuals in the top 0.1% risk tier from the pre–REACH VET period. Individuals in top 0.1% risk tiers were younger than sub-threshold cohorts in both periods (Table 1). Individuals in the REACH VET era were older than those in the earlier period. Top 0.1% risk tiers had higher proportions of men than subthreshold cohorts. Pre–REACH VET cohorts had higher proportions of men than REACH VET–era cohorts (Table 1).

**Table 1.** Characteristics of Study Cohorts

Risk tier	No.	Age, mean (SD)^b^	Sex, No. (%)^a^	
			Men	Women
REACH VET (3/2017-12/2018)				
Top 0.1%	40 816	49.8 (14.7)	37 964 (93.01)	2852 (6.99)
Top 0.3%-0.1%	48 779	52.5 (15.0)	45 112 (92.48)	3667 (7.52)
Subcohort (3/2017-6/2018)				
Top 0.1%	30 288	49.8 (14.7)	28 278 (93.36)	2010 (6.64)
Top 0.3%-0.1%	38 515	52.7 (14.9)	35 846 (93.07)	2669 (6.93)
Pre–REACH VET (3/2014-12/2015)				
Top 0.1%	36 604	49.4 (14.4)	34 398 (93.97)	2206 (6.03)
Top 0.3%-0.1%	47 114	51.6 (14.5)	43 790 (92.94)	3324 (7.06)
Subcohort				
Top 0.1%	36 602	49.4 (14.4)	34 396 (93.97)	2206 (6.03)
Top 0.3%-0.1%	47 110	51.6 (14.5)	43 787 (92.95)	3323 (7.05)

Abbreviation: REACH VET, Recovery Engagement and Coordination for Health–Veterans Enhanced Treatment.

^a^ Top 0.1% tiers had higher proportions of men than the subthreshold cohorts (Fisher exact test: pre–REACH VET, *P* < .001; REACH VET, *P* = .002). Pre–REACH VET cohorts had higher proportions of men than the REACH VET-era cohorts (Fisher exact test: Top 0.1%, *P* < .001; Top 0.3%-0.1%, *P* = .006).

^b^ Individuals in top 0.1% tiers were younger than those in sub-threshold cohorts (*t* test *P* < .001), in both time periods. Individuals in the REACH VET era were older than in the earlier period, for both the top 0.1% and subthreshold cohorts (*t* test *P* < .001).

REACH VET was implemented at all VHA facilities. From March 2017 to December 2018, program performance increased: coordinator acknowledgment that individuals were identified for REACH VET increased from 5484 of 6334 individuals (86.6%) to 6485 of 6624 individuals (97.9%); clinician acknowledgment increased from 3200 of 6334 individuals (50.5%) to 6008 of 6624 individuals (90.7%); and care evaluations increased from 2923 of 6334 individuals (46.1%) to 5796 of 6624 individuals (87.5%). Outreach attempt prevalence increased from 2736 of 6334 individuals (43.2%) to 5665 of 6624 individuals (85.5%), but successful outreach attempts decreased from 2024 of 2736 individuals for whom outreach was attempted (74.0%) to 4020 of 5665 individuals for whom outreach was attempted (71.0%).

eTable 1 and eTable 2 in the Supplement provide detail regarding 6-month outcomes for the pre–REACH VET period compared with during the REACH VET program. Table 2 presents TD results. Accounting for potential period and cohort differences and adjusting for age and sex, REACH VET identification was associated with having more completed outpatient appointments (adjusted TD [ATD], 0.31; 95% CI, 0.06 to 0.55) and lower proportions of scheduled outpatient appointments that were missed (1% reduced; ATD, −0.01; 95% CI, −0.02 to −0.01). We did not observe significant program associations with scheduled outpatient appointments or outpatient mental health visit volume. REACH VET was associated with reduced inpatient mental health admissions (ATD, −0.08; 95% CI, −0.10 to −0.05) and ED visit days (ATD, −0.03; 95% CI, −0.06 to −0.01).

**Table 2.** Results of TD Analyses for Treatment Engagement, Health Care Utilization, Suicide Attempts, and Any Suicide Prevention Safety Plan Documentation

Outcome	REACH VET Era			Pre–REACH VET Era			TD, mean (95% CI)^b^	
	Risk tier, mean change (SD), No.^a^		Difference-in-difference , mean (95% CI)^c^	Risk tier, mean change (SD), No.^a^		Difference-in-difference, mean (95% CI)^c^		
	Top 0.1%	Top 0.3%-0.1%		Top 0.1%	Top 0.3%-0.1%		Unadjusted	Adjusted for age and sex
Scheduled outpatient appointments	5.30 (22.09)	1.96 (17.83)	3.34 (3.07 to 3.61)	6.14 (21.17)	3.13 (17.25)	3.02 (2.75 to 3.28)	0.32 (−0.05 to 0.70)	0.29 (−0.07 to 0.65)
Completed outpatient appointments	3.94 (17.61)	1.53 (14.23)	2.41 (2.20 to 2.62)	4.38 (17.10)	2.36 (13.94)	2.02 (1.80 to 2.23)	0.39 (0.09 to 0.70)	0.31 (0.06 to 0.55)
Scheduled outpatient appointments missed, proportion^d^	−0.00 (0.32)	0.01 (0.31)	−0.01 (−0.02 to −0.01)	0.01 (0.33)	0.01 (0.32)	−0.00 (−0.01 to 0.00)	−0.01 (−0.02 to −0.01)	−0.01 (−0.02 to −0.01)
Outpatient mental health visits	4.73 (55.67)	−3.09 (43.18)	7.81 (7.15 to 8.48)	7.42 (51.46)	0.15 (40.65)	7.27 (6.63 to 7.92)	0.54 (−0.38 to 1.46)	0.72 (−0.43 to 1.87)
Inpatient mental health admissions	−0.77 (1.14)	−0.55 (0.84)	−0.22 (−0.24 to −0.21)	−0.72 (1.09)	−0.57 (0.83)	−0.16 (−0.17 to −0.15)	−0.06 (−0.08 to −0.05)	−0.08 (−0.10 to −0.05)
ED visit days	−0.88 (2.55)	−0.86 (1.88)	−0.03 (−0.06 to 0.00)	−0.78 (2.36)	−0.80 (1.84)	0.02 (−0.01 to 0.05)	−0.05 (−0.09 to −0.01)	−0.03 (−0.06 to −0.01)
With any documented suicide attempt, proportion	−0.10 (0.36)	−0.04 (0.22)	−0.06 (−0.07 to −0.06)	−0.07 (0.34)	−0.03 (0.22)	−0.04 (−0.05 to −0.04)	−0.02 (−0.03 to −0.02)	−0.05 (−0.06 to −0.03)
With any safety plan documentation, proportion	−0.40 (0.60)	−0.29 (0.53)	−0.11 (−0.11 to −0.10)	−0.31 (0.59)	−0.23 (0.50)	−0.09 (−0.09 to −0.08)	−0.02 (−0.03 to −0.01)	−0.01 (−0.02 to 0.01)

Abbreviations: ED, emergency department; REACH VET, Recovery Engagement and Coordination for Health–Veterans Enhanced Treatment; TD, triple difference.

^a^ Mean of change scores (subsequent 6 months − prior 6 months).

^b^ REACH VET era − pre–REACH VET era.

^c^ Mean difference calculated as top 0.1% − top 0.3% to 0.1%.

^d^ Cohorts were limited to those with at least 1 appointment in the prior and subsequent 6 months, for a total of 158 346 individuals.

REACH VET identification was associated with reduced likelihood of suicide attempt documentation in the subsequent 6 months. Accounting for potential period and cohort differences, REACH VET was associated with a 5% reduction in the probability of documentation of a suicide attempt, adjusting for age and sex (ATD, −0.05; 95% CI, −0.06 to −0.03). In unadjusted TD analyses, individuals identified by REACH VET were less likely to have documented suicide safety plans in the subsequent 6 months compared with the prior 6 months (TD, −0.02; 95% CI, −0.03 to −0.01), but the difference was no longer significant when adjusted for age and sex.

Individuals in REACH VET were more likely to have new suicide safety plans within 6 months (Table 3). Accounting for period and cohort differences and age and sex, REACH VET was associated with an increase in the probability of receiving a new suicide safety plan (ATD, 0.08; 95% CI, 0.06 to 0.10). Sensitivity analyses using 3-month periods generated consistent findings, except that analyses for proportion with safety plan documentation was statistically significant.

**Table 3.** Difference-in-Differences Analyses for Proportion of Individuals With a New Suicide Prevention Safety Plan Documentation and Mortality Outcomes in Subsequent 6 Months

Outcome	REACH VET era			Pre–REACH VET era			Difference-in-difference, mean (95% CI)^a^	
	Risk tier, mean (SD), proportion		Difference, mean (95% CI)^b^	Risk tier, mean (SD), proportion		Difference, mean (95% CI)^b^	Unadjusted	Adjusting for age and sex
	Top 0.1%	Top 0.3%-0.1%		Top 0.1%	Top 0.3%-0.1%			
With a new safety plan^c^	0.1680 (0.3739)	0.0558 (0.2295)	0.1123 (0.1046 to 0.1199)	0.1284 (0.3345)	0.0523 (0.2226)	0.0761 (0.0702 to 0.0820)	0.0362 (0.0266 to 0.0458)	0.0768 (0.0561 to 0.0974)
All-cause mortality^d^	0.0217 (0.1456)	0.0317 (0.1752)	−0.0100 (−0.0121 to −0.0079)	0.0195 (0.1381)	0.0286 (0.1668)	−0.0092 (−0.0113 to −0.0071)	−0.0009 (−0.0038 to 0.0021)	0.0000 (−0.0001 to 0.0001)
Cause-specific mortality^e^								
Suicide	0.0028 (0.0526)	0.0016 (0.0395)	0.0012 (0.0005 to 0.0019)	0.0025 (0.0501)	0.0018 (0.0427)	0.0007 (0.0000 to 0.0013)	0.0005 (−0.0004 to 0.0015)	0.0007 (−0.0006 to 0.0019)
Nonsuicide external cause	0.0052 (0.0720)	0.0043 (0.0651)	0.0010 (−0.0001 to 0.0020)	0.0038 (0.0617)	0.0030 (0.0546)	0.0008 (0.0000 to 0.0016)	0.0001 (−0.0012 to 0.0014)	0.0001 (−0.0015 to 0.0018)
All-cause^f^	0.0223 (0.1476)	0.0320 (0.1760)	−0.0097 (−0.0121 to −0.0073)	0.0192 (0.1371)	0.0283 (0.1659)	−0.0092 (−0.0112 to −0.0071)	−0.0005 (−0.0037 to 0.0026)	0.0001 (−0.0001 to 0.0002)

^a^ REACH VET era − pre–REACH VET Era.

^b^ Mean difference (top 0.1% − top 0.3% to 0.1%).

^c^ Cohorts were limited to those with no safety plan in the prior 2 years, for a total of 77 625 individuals.

^d^ Mortality ascertained from the Vital Status File. Cohorts were limited to those for whom vital status could be ascertained, for a total of 173 305 individuals.

^e^ Mortality ascertained from the Mortality Data Repository. Cohorts were limited to those for whom cause of death could be ascertained, for a total of 152 515 individuals.

^f^ Adjusted analyses used a modified Poisson model.

REACH VET was not associated with all-cause mortality within 6 months, per the VHA Vital Status File (age- and sex-adjusted difference-in-difference[ADiD], <0.0001; 95% CI, −0.0001 to 0.0001). Subcohort analyses using death certificate data through 2018 did not identify significant program associations with suicide (ADiD, 0.0007; 95% CI, −0.0006 to 0.0019), nonsuicide external cause (ADiD, 0.0001; 95% CI, −0.0015 to 0.0018), or all-cause mortality (ADiD, 0.0001; 95% CI, −0.0001 to 0.0002). Table 4 provides information regarding 6-month mortality for the REACH VET and concurrent subthreshold subcohorts and the pre–REACH VET top 0.1% and subthreshold cohorts.

**Table 4.** All-Cause, Suicide, and Nonsuicide External Cause Mortality at 6 Months Stratified by Cohort^a^

6-Month mortality	Deaths, No. (%)			
	REACH VET^b^		Pre–REACH VET^c^	
	Top 0.1% (n=30 288)	Subthreshold (0.3%-0.1%) (n=38 515)	Top 0.1% (n=36 602)	Subthreshold (top 0.3%-0.1%) (n = 47 110)
All-cause	675 (2.23)	1232 (3.20)	701 (1.92)	1334 (2.83)
Suicide	84 (0.28)	60 (0.16)	92 (0.25)	86 (0.18)
Nonsuicide external cause	158 (0.52)	164 (0.43)	140 (0.38)	141 (0.30)

Abbreviation: REACH VET, Recovery Engagement and Coordination for Health–Veterans Enhanced Treatment.

^a^ Excludes 2 individuals from the pre–REACH VET Era Top 0.1% group and 4 individuals from the pre–REACH VET era subthreshold cohort for whom mortality data were not available from the Mortality Data Repository.

^b^ Includes individuals who entered risk tier during March 2017 to June 2018.

^c^ Includes individuals who entered risk tier during March 2014 to December 2015.

## Discussion

This cohort study found that REACH VET was associated with increased completed outpatient appointments; reduced missed appointments, inpatient mental health admissions, and ED visit days; greater initiation of suicide safety plans; and reductions in documented suicide attempts. This last is noteworthy, as focused clinical attention may increase VHA ascertainment of suicide attempts, in which case study findings could underestimate this association. REACH VET was not associated with changes in overall outpatient mental health encounter volume. Future studies should assess whether this lack of change results from altered patterns of health care utilization, with increased regular and coordinated treatment maintenance contacts and decreased episodic encounters.

The feasibility and utility of suicide predictive modeling has long been anticipated and debated.^20,21,22^ The VHA has completed national implementation of a clinical program using a risk prediction algorithm to identify individuals with high suicide risk for care enhancements.

### Limitations

This study has some limitations. First, cause of death data were not available for the entire cohort. Subcohort analyses assessed available data (146 223 observations). These did not identify associations, yet may be underpowered. Power analyses using Lehr equation adapted for binomial outcomes^23^ suggest that at least 430 598 observations would be required to detect a 10% reduction in 6-month suicide mortality. Future studies are warranted when more recent mortality data becomes available. More work is needed to understand site ascertainment of nonfatal suicide attempts. It was not possible to test whether individual program components were associated with study outcomes. Additionally, while outcomes were assessed for 6-month periods, considering alternative periods may be helpful.

## Conclusions

This cohort study evaluated multiple aspects of program implementation and effectiveness of REACH VET, the nation’s first clinical implementation of a validated algorithm to support suicide risk identification. Results document the dynamic nature of health care utilization in the nation’s largest integrated health system, requiring TD analyses to adjust for nonparallel trends. Understanding impacts of the REACH VET program has clinical, scientific, and public policy significance, and REACH VET has received substantial public and Congressional attention.^24,25^

Since the development of the original VA suicide prediction model in 2015,^8^ there has been greater acceptance that electronic health records may contribute important tools for improved suicide risk identification to support care enhancement.^26^ Implementation of the REACH VET program has been substantial and program inclusion was associated with greater treatment engagement and safety plan documentation, fewer inpatient mental health admissions and emergency department visit days, and reduced prevalence of nonfatal suicide attempts. REACH VET represents a promising intervention that enhances care. The program is the direct result of long-term VA investment^1,2,3,5,8,9,10,16^ in suicide surveillance, data analytics, and clinical operations.

## Notes

Supplement. **eAppendix.** Parallel-Trend Assumption Tests for REACH VET Evaluation Outcomes Prior to and After Identification **eTable 1.** Mean Continuous Outcomes per Cohort and Time Period **eTable 2.** Proportion Outcomes per Cohort and Time Period
