# Supplementary material for: Evaluation of the Recovery Engagement and Coordination for Health–Veterans Enhanced Treatment Suicide Risk Modeling Clinical Program in the Veterans Health Administration
Source: JAMA Netw Open. 2021 Oct 18;4(10):e2129900. doi: 10.1001/jamanetworkopen.2021.29900 (PMC8524305; doi:10.1001/jamanetworkopen.2021.29900)
Supplement: Supplement. — eAppendix. Parallel-Trend Assumption Tests for REACH VET Evaluation Outcomes Prior to and After Identification eTable 1. Mean Continuous Outcomes per Cohort and Time Period eTable 2. Proportion Outcomes per Cohort and Time Period [file jamanetwopen-e2129900-s001.pdf]

Supplemental Online Content

McCarthy JF, Cooper SA, Dent KR, et al. Evaluation of the Recovery Engagement and Coordination for Health–Veterans Enhanced Treatment suicide risk modeling clinical program in the Veterans Health Administration. *JAMA Netw Open*. 2021;4(10):e2129900. doi:10.1001/jamanetworkopen.2021.29900

eAppendix. Parallel-Trend Assumption Tests for REACH VET Evaluation Outcomes Prior to and After Identification

eTable 1. Mean Continuous Outcomes per Cohort and Time Period

eTable 2. Proportion Outcomes per Cohort and Time Period

This supplemental material has been provided by the authors to give readers additional information about their work.

© 2021 McCarthy JF et al. *JAMA Network Open.*

eAppendix. Parallel-Trend Assumption Tests for REACH VET Evaluation Outcomes Prior to and After Identification

Background

Difference in differences analyses assume that in the absence of an intervention the differences between treatment and control groups would remain constant over time.[1] In cases where parallel trends are not observed in a pre-intervention period, it is appropriate to employ a triple differences (TD) study design, in order to adjust for non-parallel trends. In this study, the parallel trend assumption was assessed for each measure, for two possible differences-in-differences approaches: 1) differences across REACH VET and pre-REACH VET time periods among patients identified in the top 0.1% risk tier, and 2) differences across risk tiers (top 0.1% versus subthreshold) among those in the REACH VET era. Panel a and b in the illustrative figure below show violations of the parallel trend assumption, thus requiring a TD approach, and panel c demonstrates parallel trends, which would be consistent with use of the difference-in-differences approach.

Appendix Figure: Parallel Trends Assumption and Implications for Design: Illustrative Examples

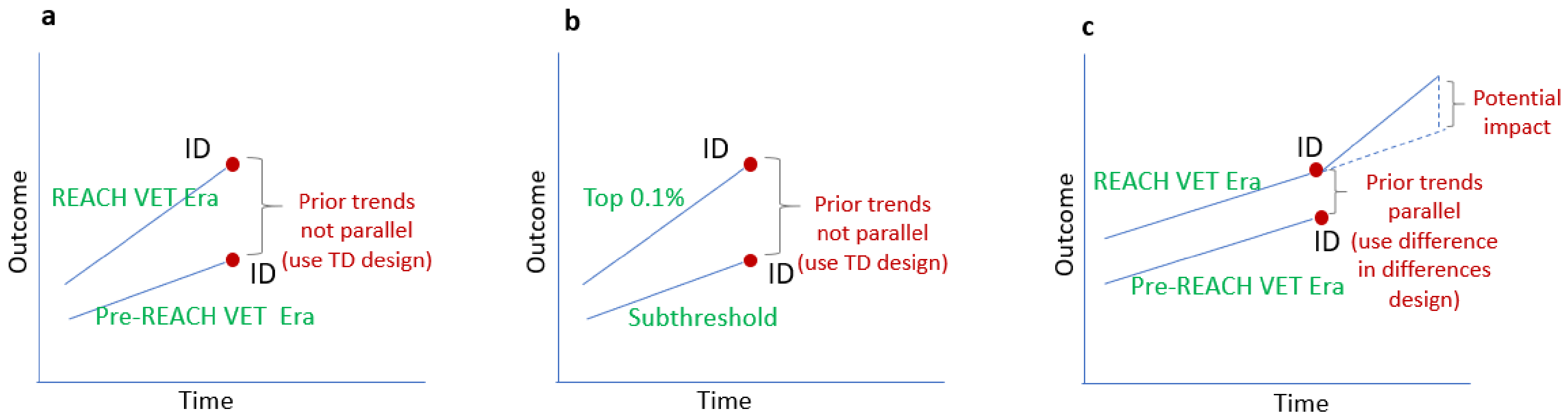

© 2021 McCarthy JF et al. *JAMA Network Open.*

Methods

To assess for parallel trends, outcomes were collected across four 6-month periods prior to each patient's cohort entry (prior 0-6 months, prior 6-12 months, prior 12-18 months, prior 18-24 months). The parallel trend assumption across REACH VET eras among individuals in the top 0.1% risk tier was assessed using an interaction term between time period and REACH VET era (Model 1). The parallel trend assumption across REACH VET risk tiers among those in the REACH VET era was assessed using an interaction between time period and REACH VET risk tier (Model 2). The estimate and 95% confidence intervals for the difference in the associations with time across groups are presented in Appendix Table 1, below. A 95% confidence interval excluding zero indicates a statically significant difference in the pre-identification trends across groups during the 24 months prior to identification into the REACH VET cohort. In these cases, the parallel trend assumption is violated.

Model 1: Parallel Trend Assumption Test for Differences in Differences across REACH VET Eras among the Top 0.1% Risk Tier

$$\text{Outcome}=\beta_0+\beta_1\text{*REACH\_VET\_Era}+\beta_2\text{*Time}+\beta_3\text{*REACH\_VET\_Era*Time}+\varepsilon_i$$

Population was limited to those in the top 0.1% risk tier.

Time was a continuous indicator for the four 6-month periods prior to the patient's REACH VET identification date.

Model 2: Parallel Trend Assumption Test for Differences in Differences across REACH VET Risk Tiers in the REACH VET Era

$$\text{Outcome}=\beta_0+\beta_1\text{*Top0.1\%Tier}+\beta_2\text{*Time}+\beta_3\text{* Top0.1\%Tier *Time}+\varepsilon_i$$

Population was limited to those in the REACH VET era.

Time was a continuous indicator for the 4, 6-month periods prior to the patients REACH VET identification date.

© 2021 McCarthy JF et al. *JAMA Network Open.*

While the triple differences estimation adjusts for non-parallel trends across groups, there may be residual differences in trends across comparison groups.[1] To assess any differences in trends within the pre-identification period, we used an interaction between time period, REACH VET risk tier, and REACH VET era (Model 3). Estimates with a 95% confidence interval that did not include zero indicated that adjustment for period and risk-tier was insufficient to eliminate all pre-REACH VET identification trends. There may be unobservable factors contributing to trends in these outcomes even in the absence of the REACH VET program and consequently results for outcomes that violate the triple differences parallel trend assumption should be interpreted with caution.

© 2021 McCarthy JF et al. *JAMA Network Open.*

Model 3: Parallel Trend Assumption for Triple Differences

$$Outcome=\beta_0+\beta_1*Time+\beta_2*REACH\_VET\_Era+\beta_3*Top0.1\% +\beta_4*Time*REACH\_VET\_Era+\beta_5*Time*Top0.1\%$$

$$+\beta_6*REACH\_VET\_Era*Top0.1\%+\beta_7*Time*REACH\_VET\_Era*Top0.1\%+\varepsilon_i$$

Time was a continuous indicator for the four 6-month periods prior to the patients REACH VET identification date.

Results

eAppendix Table. Parallel Trend Assumption Tests[a]

	Differences in Differences across REACH VET Eras within Top 0.1% Risk Tier	Differences in Differences across Threshold Groups within the REACH VET Era	Triple Differences
Outcome	Estimate (95% Confidence Interval)	Estimate (95% Confidence Interval)	Estimate (95% Confidence Interval)
Average Scheduled Outpatient Appointments	-0.023 (-0.082, 0.037)	**0.136 (0.075, 0.197)**	0.005 (-0.075, 0.086)
Average Completed Outpatient Appointments	-0.031 (-0.080, 0.018)	0.015 (-0.034, 0.065)	-0.028 (-0.095, 0.037)
Proportion of Scheduled Outpatient Appointments Missed	-0.002 (-0.004, 0.001)	**0.013 (0.011, 0.016)**	0.003 (-0.001, 0.006)
Average Outpatient Mental Health Visits	**0.610 (0.528, 0.691)**	**1.478 (1.381, 1.575)**	**0.531 (0.409, 0.652)**
Average Inpatient Mental Health Admissions	**0.016 (0.014, 0.019)**	**0.040 (0.039, 0.042)**	**0.019 (0.017, 0.022)**
Average Emergency Department Visit Days	**0.020 (0.016, 0.025)**	**0.064 (0.059, 0.069)**	**0.016 (0.010, 0.022)**
Proportion with Any Documented Suicide Attempt	**0.002 (0.002, 0.003)**	**0.005 (0.004, 0.005)**	**0.002 (0.001, 0.003)**
Proportion with Any Safety Plan Documentation	**0.017 (0.016, 0.018)**	**0.015 (0.014, 0.016)**	**0.007 (0.006, 0.009)**

[a] bold indicates significance and violation of parallel trends assumption.

The parallel trend assumption was violated for all differences-in-differences analyses except for average number of scheduled outpatient appointments, average number of completed outpatient appointments, and proportion of scheduled outpatient appointments missed across REACH VET eras, among the top 0.1% risk

© 2021 McCarthy JF et al. *JAMA Network Open.*

tier, and for number of completed outpatient appointments across risk tiers, in the REACH VET era. In these instances, a differences-in-differences approach may have been sufficient, yet since all other outcomes indicated that a TD analysis was warranted, that more conservative approach was employed for all outcomes.

The TD parallel trend assumption was violated for all outcomes except the number of scheduled and completed outpatient appointments and the proportion of scheduled outpatient appointments that were missed. The violation of the TD parallel trend assumption does not prevent one from drawing conclusions, yet requires a more nuanced interpretation of the results.[1,2,3,4] Trends for inpatient mental health admissions, emergency department visit days, prevalence of any documented suicide attempt, and prevalence of any safety plan documentation prior to identification into REACH VET were in the opposite direction of the observed associations of the REACH VET program. This suggests that the true magnitude of the REACH VET association may be larger than what was documented, given the presence of non-parallel trends. Meanwhile, the pre-identification trend for outpatient mental health visits was in the same direction as the REACH VET association. However, since the TD assessment of the REACH VET association with outpatient mental health visits was non-significant, the pre-identification trend does not change our conclusions. Overall, pre-identification trends were close to zero and frequently were in the opposite direction from the observed REACH VET TD associations. The latter case suggests that there may be greater REACH VET associations than were ascertained in the TD analysis, because the TD design insufficiently controlled for pre-existing trends in the outcome. We note that significant TD parallel trend assumption tests may reflect the high statistical power of these tests rather than substantive differences in trends.

[1]Olden A, Moen J. The Triple Difference Estimator. NHH Dept. of Business and Management Science Discussion Paper No. 2020/1. 2020. Retrieve from https://ssrn.com/abstract=3582447

[2]Chen Q, Chu X, Wang S, Zhang B. A Triple-Difference Approach to Re-Evaluating the Impact of China's New Cooperative Medical Scheme on Incidences of Chronic Diseases Among Older Adults in Rural Communities. Risk Manag Healthc Policy. 2020;13:643-659. Published 2020 Jun 24. doi:10.2147/RMHP.S244021

© 2021 McCarthy JF et al. *JAMA Network Open.*

[3]Keng SH, Sheu SJ. The effect of national health insurance on mortality and the SES-health gradient: evidence from the elderly in Taiwan. Health Econ. 2013;22(1):52-72. doi:10.1002/hec.1815

[4]Kim S, Kwon S. The effect of extension of benefit coverage for cancer patients on health care utilization across different income groups in South Korea. Int J Health Care Finance Econ. 2014;14(2):161-177. doi:10.1007/s10754-014-9144-y

© 2021 McCarthy JF et al. *JAMA Network Open.*

eTable 1. Mean Continuous Outcomes per Cohort and Time Period

	Pre-REACH Vet Top 0.1%				Pre-REACH Vet 0.3%-0.1%				REACH Vet Top 0.1%				REACH Vet 0.3%-0.1%			
	Prior 6-Months		Subsequent 6-Months		Prior 6-Months		Subsequent 6-Months		Prior 6-Months		Subsequent 6-Months		Prior 6-Months		Subsequent 6-Months	
Outcome	Average	Std Dev	Average	Std Dev	Average	Std Dev	Average	Std Dev	Average	Std Dev	Average	Std Dev	Average	Std Dev	Average	Std Dev
Scheduled Outpatient Appointments	10.70	13.1	16.85	21.6	10.69	12.9	13.82	17.7	11.82	14.8	17.12	22.3	11.62	14.5	13.58	18.0
Completed Outpatient Appointments	6.93	9.9	11.31	17.6	7.33	10.0	9.69	14.4	7.56	11.3	11.50	17.8	8.00	11.2	9.54	14.2
Outpatient Mental Health Visits	30.23	32.2	37.66	52.6	23.51	31.9	23.66	41.6	36.01	37.8	40.74	56.8	26.15	36.1	23.06	43.1
Inpatient Mental Health Admissions	1.22	0.8	0.49	1.0	0.78	0.8	0.21	0.6	1.27	0.9	0.50	1.0	0.74	0.8	0.19	0.6
Emergency Department Visit Days	2.08	2.3	1.30	2.4	1.70	2.0	0.90	1.8	2.21	2.6	1.32	2.6	1.72	2.1	0.87	1.8

© 2021 McCarthy JF et al. *JAMA Network Open.*

eTable 2. Proportion Outcomes per Cohort and Time Period

	Pre-REACH Vet Top 0.1%		Pre-REACH Vet 0.3%-0.1%		REACH Vet Top 0.1%		REACH Vet 0.3%-0.1%	
	Prior 6-Months	Subsequent 6-Months	Prior 6-Months	Subsequent 6-Months	Prior 6-Months	Subsequent 6-Months	Prior 6-Months	Subsequent 6-Months
Outcome	N(%)	N(%)	N(%)	N(%)	N(%)	N(%)	N(%)	N(%)
Scheduled Outpatient Appointments Missed[a]	135,803 (35.0)	194,896 (32.7)	154,785 (31.2)	188,312 (29.7)	170,886 (35.9)	224,649 (32.8)	170,924 (30.8)	193,795 (29.7)
Any Documented Suicide Attempt	3,778 (10.3)	1,040 (2.8)	2,049 (4.3)	468 (1.0)	5,251 (12.9)	1,188 (2.9)	2,214 (4.5)	405 (0.8)
Any Safety Plan Documentation	19,508 (53.3)	7,992 (21.8)	15,774 (33.5)	4,982 (10.6)	28,021 (68.7)	11,778 (28.9)	20,430 (41.9)	6,196 (12.7)
New Safety Plan[b]	---	1,982 (12.8)	---	1,439 (5.2)	---	1,812 (16.8)	---	1,332 (5.6)
All-Cause Mortality[c]	---	712 (1.9)	---	1,349 (2.9)	---	884 (2.2)	---	1,546 (3.2)

[a]Total appointments missed. Cohorts were limited to those with at least one appointment in the prior and subsequent six months (Total N = 158,346).

[b]Cohorts were limited to those with no safety plan in the prior two years (Total N = 77,625)

[c]Mortality ascertained from the Vital Status File. Cohorts were limited to those for which vital status could be ascertained (Total N = 173,305)

© 2021 McCarthy JF et al. *JAMA Network Open.*
